# Probiotics for Standard Triple *Helicobacter pylori* Eradication: A Randomized, Double-blind, Placebo-controlled Trial

**DOI:** 10.1097/MD.0000000000000685

**Published:** 2015-05-01

**Authors:** Goran Hauser, Nermin Salkic, Karina Vukelic, Alenka JajacKnez, Davor Stimac

**Affiliations:** From the Department of Gastroenterology, Clinical Hospital Centre, Rijeka, Rijeka, Croatia (GH, DS); Department of Gastroenterology and Hepatology, University Clinical Centre, Tuzla, Tuzla, Bosnia and Herzegovina (NS); and JGL d.d. Rijeka, Croatia (KV, AJ).

## Abstract

The primary objective in the study is determination of efficacy of probiotic preparation as a supportive therapy in eradication of *Helicobacter pylori*.

The study was multicenter, prospective, randomized, placebo controlled, and double-blind. The subjects first filled out a specially designed questionnaire to assess the severity of the 10 symptoms, which can be related to eradication therapy to be monitored during the trial. Each subject then received 28 capsules of probiotic preparation or matching placebo capsules, which they were supposed to take over the following 14 days, twice a day, at least 2 hours prior to or after the antibiotic therapy administration.

A total of 804 patients were enrolled in the trial, of which 650 (80.85%) were included in the analysis. The results show a significantly larger share of cured subjects in the probiotic arm versus the placebo arm (87.38% vs 72.55%; *P* < 0.001). Additionally, presence and intensity of epigastric pain, bloating, flatulence, taste disturbance, loss of appetite, nausea, vomiting, heartburn, rash, and diarrhea were monitored over the study period. At 15 days postinclusion, probiotic treatment was found superior to placebo in 7 of 10 mentioned symptoms. Average intensity for symptoms potentially related to antibiotic therapy was significantly higher in the placebo group, 0.76 vs 0.55 (*P* < 0.001).

Adding probiotics to the standard triple therapy for *H pylori* eradication significantly contributes to treatment efficacy and distinctly decreases the adverse effects of therapy and the symptoms of the underlying disease.

## INTRODUCTION

The term probiotic essentially signifies a substance opposite to antibiotic. The concept of probiotic is based on the articles of Russian scientist Ilya Ilyich Mechnikov,^1^ who noticed that certain strains of *Lactobacillus* have the ability to eliminate pathogenic bacteria from the gastrointestinal system.

Even at a glance, the literature reveals a significant number of clinical studies on the use of probiotics,^2^ especially in the gastrointestinal indications such as antibiotic-induced diarrhea,^3–8^ other types of diarrhea like the so-called “traveller's diarrhea,”^9–12^ and gastroenteritis in children^13–17^ and adults.^18,19^

In line with these findings, gastroenterologists and general practitioners have increasingly more possibilities of prescribing probiotic preparations as the only or (more often) adjuvant therapy in certain indications. A significant step forward was made in the current report of the European Helicobacter Study Group,^20^ which considers probiotics as an adjuvant treatment in reducing side-effects during the standard *Helicobacter pylori* (*H. pylori*) eradication therapy. At this stage, probiotics are classified as Grade D recommendation and that is the reason why clinical studies are necessary for such effects to be objectively proven in a clinical setting.

The role of probiotics in such treatment is to reduce the number and intensity of side-effects and to act as an adjuvant to standard treatment, resulting in better patient compliance and better treatment outcome.

We aimed to conduct a clinical study for *H pylori* eradication using probiotics as an adjunctive treatment to standard triple therapy regimen. The primary outcome of the study was efficacy of eradication therapy and the secondary outcomes were possible improvement of side-effects and tolerability of eradication therapy by adding the probiotic preparation.

## MATERIALS/METHODS

### Study Population and Design

The trial was prospective, randomized, placebo-controlled, double-blind, multicenter trial. It was conducted according to the Declaration of Helsinki Principles and was approved by the institutional review board. This study was performed in compliance with Good Clinical Practices, including the archiving of essential documents. The study was reported according to the CONSORT guidelines and was registered at www.clinicaltrials.gov (NCT01969331). Informed consent for this study was obtained from all patients. Inclusion criteria were: confirmed *H pylori* infection, otherwise healthy subjects taking *H pylori* eradication therapy, age above 18 years irrespective of sex, and subjects who provided written informed consent prior to undergoing any study procedure. Exclusion criteria were: pregnancy or lactation; severe diseases such as malignant diseases, decompensated renal, cardiac, pulmonary or liver illness; subject who is not mentally capable of adhering to the protocol; drug addiction or alcoholism; any other clinical condition which, in the opinion of the attending physician, would not allow safe administration of the study medications; and subjects participating in any other clinical trial.

Probiotic preparation contains *Lactobacillus rhamnosus* GG (LGG^®^) and *Bifidobacterium (*BB-12^®^) in the concentration of 10^8^ to 10^10^ living bacteria capable of reproduction per capsule (Normia^®^, JGL, Croatia, Christian Hanssen, Denmark).

The enrolment of subjects into the trial was conducted in 121 general practitioners’ offices, in different regions in Croatia from December 2008 until December 2010.

The initial diagnosis of *H pylori* infection was established using 1 of 3 commonly accepted methods—rapid urease test, stool antigen, or urea breath test.^21^ After obtaining the informed consent form, the subjects were enrolled in the trial and randomized either to the placebo arm or to the probiotic arm. All patients received standard triple *H pylori* eradication therapy.

Randomization was centrally conducted using standard methodology with a computer-generated list.^22^ After assignment, the participants and care providers (general practitioners) were blinded to interventions. The subjects first filled out a specially designed questionnaire to assess the severity of each of the 10 symptoms, which can be related to eradication therapy to be monitored during the trial. Each subject then received 28 capsules of probiotic preparation or matching placebo capsules, which they were supposed to take over the following 14 days, twice a day, at least 2 hours prior to or after the antibiotic therapy administration. Considered as nonadherent were subjects who took <80% of either standard therapy or probiotic. Compliance was monitored by counting the remaining medication at the next study visit. The most common combinations of the eradication therapy were omeprazole (2 × 20 mg) or pantoprazole (2 × 40 mg) + clarithromycin (2 × 500 mg) + amoxicillin (2 × 1000 mg), followed by same combination but with metronidazole (2 × 400 mg) instead of amoxicillin. A number of other treatments including lansoprazole as proton pump inhibitor (PPI) (2 × 30 mg) and azithromycin as antibiotic as well as other combinations were responsible for the remaining 9% of study treatments. Each study center was responsible for deciding what combination to use.

**Figure 1:**
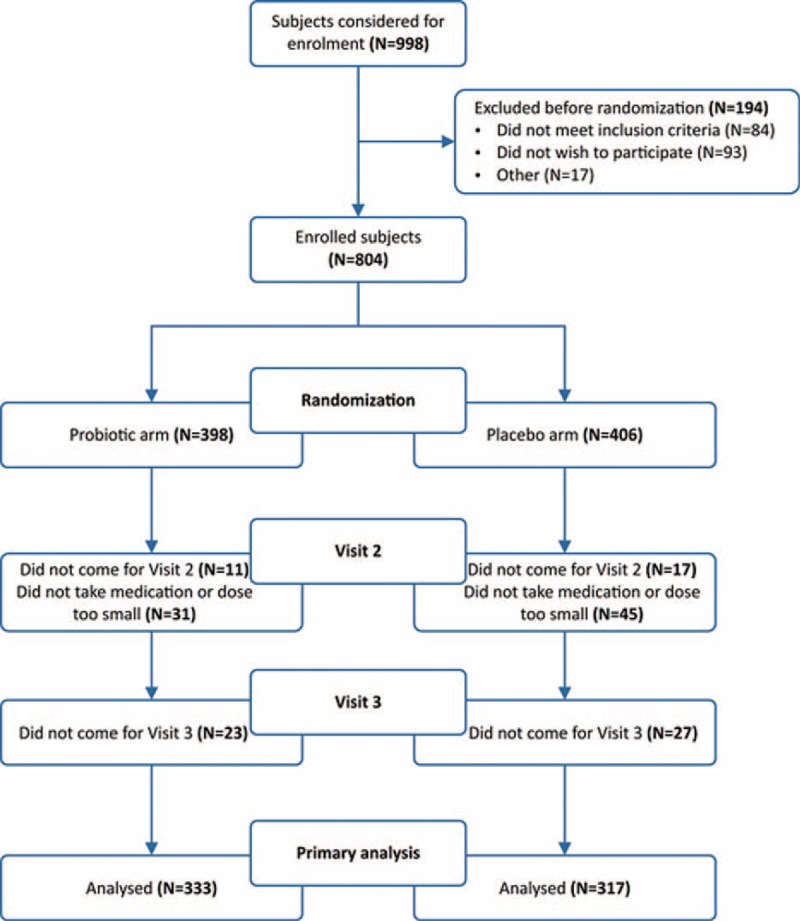
Comparison of treatment arms at 15 days, according to monitored symptoms.

In addition to the described initial visit, the subjects were monitored during 2 additional visits—at 15 and 42 days after enrolment. On the second visit, 15 days after the start of the trial, the subjects again assessed the intensity of the 10 monitored symptoms, as at enrolment, and their compliance was also checked (Figure 1). On the third visit, 42 days after enrolment, the diagnostic procedure to establish the presence of *H pylori* was again conducted for each subject using the same method that was used when the initial diagnosis was established except for rapid urease test wherein we used one of the noninvasive tests.

### Outcome Measures

The primary endpoint of the trial was defined as the share of subjects with successfully administered treatment, that is, negative result for *H pylori* at 6 weeks (42 days ± 2 days). The primary analysis of interest was the odds ratio (OR) for cure in the probiotic treatment arm, versus the placebo treatment arm. An additional objective was to monitor the intensity of each of the 10 symptoms using a verbal rating scale consisting of 4 categories (from 0 [no symptoms] to 3 [severe symptoms]) at baseline and at 2 weeks after enrolment.

### Sample Size Calculation and Statistical Analysis

**Figure 2:**
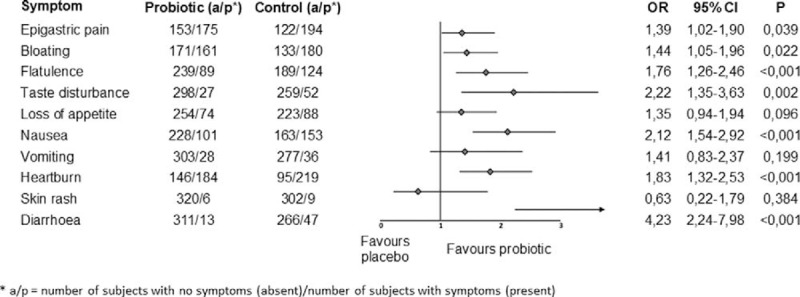
Subject distribution by arm.

The sample size was calculated using the software package NCSS/PASS^23^ to achieve appropriate statistical power (β = 0.90), with the acceptable type 1 error (α = 0.05), and hypothesis that the OR for cure in the probiotic treatment arm versus placebo is at least 2. The required sample with the aforementioned assumptions was 288 subjects per treatment arm. With expected significant withdrawal of subjects during the trial, the final number of enrolled subjects was slightly over 800 (Figure 2). The statistical analysis was conducted using the Statistica^24^ software package, with the statistical significance level set to 0.05. The values of categorical variables were shown in contingency tables and compared using the *χ*^2^ test. The values of variables measured by the interval scale were presented in descriptive values, and the differences were analyzed through appropriate parametric or nonparametric tests, depending on the distribution normality (tested by the Kolmogorov–Smirnov test). All authors had access to the study data and reviewed and approved the final manuscript.

## RESULTS

A total of 804 subjects meeting the inclusion/exclusion criteria were enrolled in the trial, of which 650 (80.85%) were included in the final analysis. As shown in Figure 2 and Table 1, the most common reason for subjects’ exclusion from the final analysis was failure to adhere to the dosage regimen prescribed by the protocol (N = 78) and failure to attend the second and/or third visit (N = 76). Table 1 further shows that at enrolment, the 2 arms were statistically comparable in all observed aspects. The primary objective of the trial was to determine the share of cured persons in the probiotic treatment arm, in comparison with the placebo treatment arm. The results in Table 2 show a significantly larger (*P* < 0.001) share of cured subjects in the probiotic arm versus the placebo arm 87.38% (95% CI = 84.33%–90.21%) vs 72.55% (95% CI = 69.65%–75.81%). Additionally, OR, absolute and relative risk reductions as well as number needed to treat all point strongly in favor of probiotic arm (Table 2). Owing to a relatively high patient drop-out rate, a worst/best analysis was performed to assess the effect of missing data (ie, subjects lost to follow-up as well as those not complying with the protocol) on the overall result. In the intention-to-treat analysis (ITT), when all subjects omitted from the analysis for the above-mentioned reasons were imputed with the “worst” outcome, that is, they were considered as not cured at the end of the study period, an OR of 2.08 (95% CI 1.53–2.83) was found. Ratios of cured subjects were 73.12% and 56.65% for probiotic and placebo arms, respectively. In the opposite case, that is, when all omitted subjects were considered as cured (ie, “best” outcome), an OR of 2.31 (95% CI 1.53–3.51) was found. Ratios of cured subjects were 87.38% and 72.55% for probiotic and placebo arms, respectively.

**Table 1:**
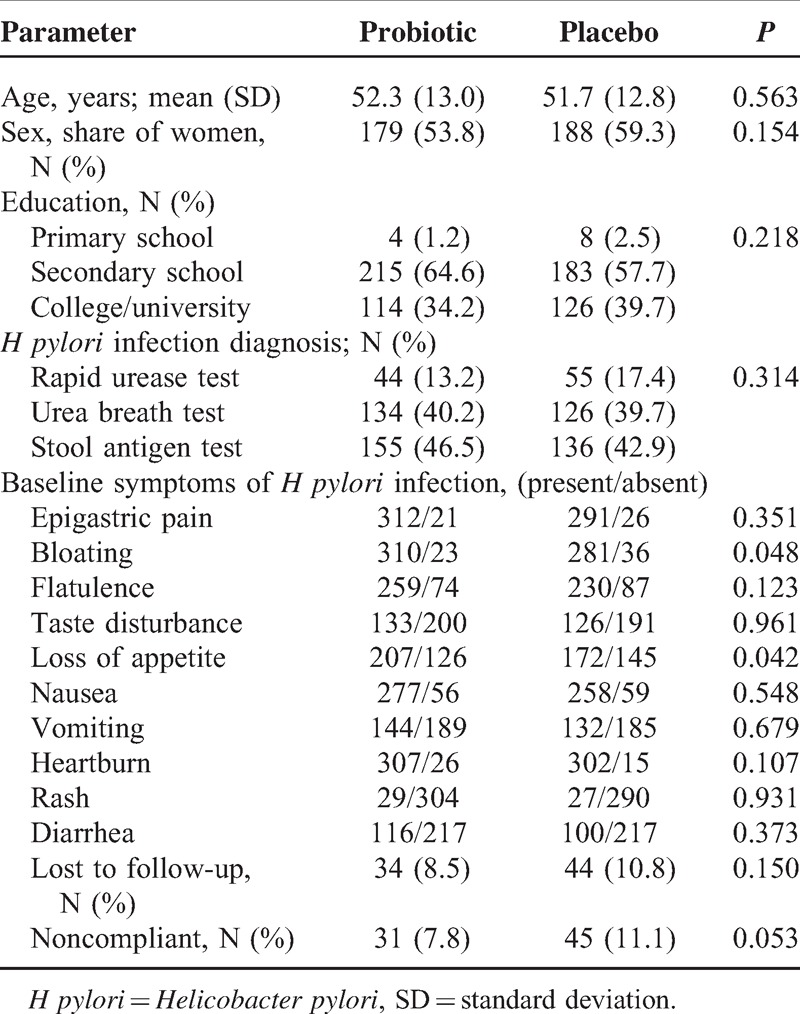
Comparison of Treatment Arms at Baseline

**Table 2:**
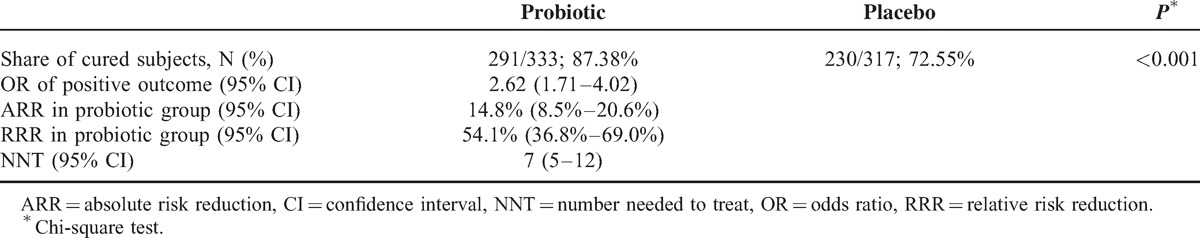
Comparison of Groups According to Share of Cured Subjects, 6 Weeks After Trial Start

Additionally to primary study outcome (ie, cured/not cured), presence and intensity for a number of symptoms (epigastric pain, bloating, flatulence, taste disturbance, loss of appetite, nausea, vomiting, heartburn, rash, and diarrhea) were monitored over the study period. The intensity was measured by the previously described scale consisting of 4 values, where the absence of symptoms was graded as 0, and the severest symptom intensity was graded as 3. At enrolment, subjects in both groups had comparable symptoms in terms of intensity, with *P* values >0.1 for all symptoms (Mann–Whitney *U* test). Same was true for the presence of symptoms (regardless of their intensity), assessed by *χ*^2^ test (after Bonferroni correction for multiple comparisons; Table 1).

In this context, Figure 1 shows presence or absence of mentioned symptoms at the follow-up visit 15 days postinclusion, in the form of ORs and forest plot. ORs show statistically significant superiority of probiotic treatment over placebo for 7 of 10 symptoms observed.

## DISCUSSIONS AND CONSLUSION

The goal of this trial was to determine objectively the efficacy of probiotics administration as adjuvant therapy in the standard approach to the treatment of *H pylori* infection. Owing to *H pylori's* immediate effects of causing gastritis and peptic ulcer as well as implications of this infection in local tumorigenesis and numerous extra intestinal manifestations, its treatment is a frequent and almost routine task of general practitioners and gastroenterologists. However, standard triple therapy (STT) approach demonstrates an ever-decreasing percentage of cured patients, recently typically <70%.^20^ This is mainly because of an increasing resistance to antibiotics (mainly clarithromycin) and is especially common when antibiotics are used in shorter administration regimens, either intentionally or because of the low compliance.^25^ Although the resistance patterns can be avoided by introduction of different antibiotics, low compliance (mainly because of adverse events) is a problem that requires a different approach.

In this context, probiotics could represent a valid support to the STT. Such combination of drugs and probiotics means that the patient still receives a certain “gold standard” in terms of therapy, while possibly benefitting from added probiotics. Probiotics address both issues that affect STT efficacy—by reducing the frequency of side-effects to antibiotic treatment,^26,27^ they increase patient compliance and by eliminating the need for additional antibiotics,^28^ they greatly reduce possibility for antibiotic resistance. Moreover, several species of probiotics have shown a direct inhibitory activity on *H pylori*,^29^ although in clinical trials, the probiotic treatment alone was not able to completely eradicate the bacterium and thus make the STT redundant.^30^

The combination of antibiotic resistance and promising results of early in-vitro^31^ studies and animal studies^32^ have resulted in a large number of clinical trials mostly in favor of using probiotics in this indication, although there are diverging opinions.^33,34^

A particular and much discussed element of the design of the present study was selection of disease-specific therapy, that is, combination of PPIs and antibiotics to be used in each individual case. According to our protocol, this decision was solely under discretion of attending physicians. We are fully aware that most authors choose entirely different method. The “usual” approach involves selecting a limited number of standard treatment combinations (in this case PPI + antibiotic), adding a novel treatment option (in this case a probiotic) and then controlling for standard treatment combinations in statistical analysis of the effect of a novel treatment option. For numerous reasons, we see such approach more as a source of bias and less as a true advantage in study design. First and most important, it is deeply unrealistic for everyday clinical or primary care setting. Patients do not receive a limited number of strictly controlled treatments but rather a wide variety of drug combinations according to their individual needs. Second, small number of treatment options can be a benefit and can be reasonably easily introduced in smaller trials. In larger trials, such as the one presented here, they pose an organizational and ethical problems. Third, although it has a clear advantage in situations wherein treatment introduced is completely novel, probiotics have already been to some degree proven to be effective in number of smaller clinical trials. In our opinion, a large trial can only benefit from the variety of different treatments. For all the mentioned reasons, we decided to omit controlling for eradication therapy and embrace a more realistic scenario of individual treatment approaches. We feel that gains in understanding of the role of probiotics in such real-life setting clearly outweigh losing some meticulousness in the statistical analysis.

With this trial, we attempted to eliminate the shortcomings observed in a considerable number of previously quoted studies. We are primarily referring to the sample size, which is very often inadequately small (or the sample calculation procedure is not even described); hence, the results are impossible to generalize so as to pertain to the general population. Meta-analyses somewhat resolve this problem, but can be deficient with regard to the heterogeneity of subject samples and the preparations administered. The meta-analysis^35^ of 14 randomized clinical trials (RCTs) with 1671 subjects included trials with 4 different probiotic strains and 6 additional combinations of probiotics. Unequal efficacy of these strains and probiotic combinations is in our opinion a severe drawback of this analysis. Other meta-analyses upon closer inspection also tend to display certain population inhomogeneity, such as inclusion of pediatric population.^36^ Most of such problems, ranging from ethnical heterogeneity (important for CYP2C19 polymorphisms) to methodological shortcomings reflected in low Jadad scores of individual clinical trials, have been reviewed in a recent article by Wang et al.^37^ Guided by such scrupulous principles, our intention was to examine a homogenous population of a sufficient and statistically validated size, with a clearly defined hypothesis and applying the identical probiotic preparation available in most markets.

Our article demonstrates that the share of successfully cured persons in the group receiving a combination of standard therapy and probiotic is statistically significantly greater than in the control group, which received STT only. In the control group, at 6 weeks post-baseline, the degree of cure was 72.55%, similar to the one described by other recent studies,^27^ which proves the hypothesis on the increased level of resistance to known antibiotics. The additional use of probiotics results in absolute risk reduction of negative outcome, that is, persistent *H pylori* infection after the end of treatment, of 14.8%.

The second important finding in this trial was also a considerably more pronounced reduction in disease symptoms in the probiotic arm, as early as at 2 weeks of treatment, that is, after the discontinuation of antibiotic administration and before the beginning of the second part of the treatment in which therapy is continued only with a PPI over 3 weeks. In the probiotic arm, ORs were in favor of probiotic treatment for all symptoms with the exception of rash. For 7 of 10 symptoms, differences between treatments were statistically significant (Figure 1).

This study had potential limitations because we included just patients at primary care setting. Furthermore, patients did not receive uniform eradication therapy protocol. However, we aimed to conduct a study that will reflect real-life setting and whose results can be applied at any general practice office. Although we stand by our initial decision to compare this novel treatment approach to “liberalized” triple therapy (as opposed to a standard and clearly defined number of treatments), following is a short discussion of therapies used. According to recent studies, percentage of clarithromycin resistance in Croatia is as high as 25.6% in continental part of the country and comparable 22% in coastal regions.^38^ However, with resistance percentage rising steeply from only 7% in 1999,^39^ many primary care physicians stick by the approach they know and understand—the standard triple therapy. In Croatia, and consequently in our study, the most common combinations were omeprazole (2 × 20 mg) or pantoprazole (2 × 40 mg) + clarithromycin (2 × 500 mg) + amoxicillin (2 x 1000 mg) in two thirds of all study subjects, followed by same combination but with metronidazole (2 × 400 mg) instead of amoxicillin in slightly less than one quarter of all subjects. A number of other treatments including lansoprazole as PPI (2 × 30 mg) and azithromycin as antibiotic as well as other combinations were responsible for the remaining 9% of study treatments. There were no treatment differences between study arms (*P* = 0,456, *χ*^2^ test).

To put our results in wider perspective and to address the issue of rationale for conducting the study in the first place, perhaps the most instructive is a recent study by Molina-Infante et al.^40^ After evaluating 9 meta-analyses of usage of probiotics as adjuvant in *H pylori* eradication, authors remain sceptic toward their widespread use for this indication. Although this is partly our opinion as well, we consider the wide variety of probiotics used (*Lactoferrin*, *Lactobacillus*, alone or combined with *Bifidobacterium*, *Saccharomyces* spp, etc) to be the source of conflicting results reported in this study and elsewhere.^41^ One of the rationales for our study was therefore to provide evidence for using a clearly defined probiotic in a realistic general care setting. In the future, we hope to conduct a study that will also clearly address effect of probiotics in different treatments. However, with levofloxacin still not being registered for this indication in Croatia and with bismuth not being on a positive list of approved drugs (ie, patients have to pay full price for bismuth preparations), the number of realistically available treatments is smaller compared with other European countries.^42^

In light of the results presented, we believe that adding probiotics to the standard triple therapy for *H pylori* infection significantly contributes to treatment efficacy and distinctly decreases the adverse effects of therapy and the symptoms of the underlying disease.
